# *Mycobacterium tuberculosis* H37Rv Short Linear PDZ-Binding Motif Proteins at the Host–Pathogen Interface

**DOI:** 10.3390/ijms27073153

**Published:** 2026-03-31

**Authors:** Edgar Sevilla-Reyes, Jorge Rosas-García, Luis Horacio Gutiérrez-González, Teresa Santos-Mendoza

**Affiliations:** Laboratory of Transcriptomics and Molecular Immunology, Instituto Nacional de Enfermedades Respiratorias Ismael Cosío Villegas, Mexico City 14080, Mexico; edgar.sevilla@iner.gob.mx (E.S.-R.); jorge.rosas@iner.gob.mx (J.R.-G.); lhgut@iner.gob.mx (L.H.G.-G.)

**Keywords:** *Mycobacterium tuberculosis*, protein–protein interaction, PDZ-binding motif, virulence factor, host–pathogen interaction, SLiMs, type VII secretion system

## Abstract

Short linear motifs (SLiMs), such as PDZ-binding motifs (PDZbms), are compact interaction modules that mediate transient, specific protein–protein interactions. While PDZbms are well characterized in viral pathogenesis, subverting host protein functions, their role in bacterial systems requires further study. *Mycobacterium tuberculosis* (Mtb) is an intracellular pathogen that mainly infects macrophages. The type VII secretion system (T7SS) of Mtb secretes a subset of effector proteins (Esx) involved in virulence. By using molecular docking and support vector machine-based prediction, we analyzed PDZbm occurrence in T7SS Esx effector proteins and their ability to bind human PDZ domain-containing proteins. We identified PDZbms in most of the Esx proteins studied, with EsxA and EsxG showing the best PDZ-dependent interaction with syntenin-1, a host scaffold protein involved in vesicular trafficking and immune signaling. Additional Esx proteins were predicted to engage other host PDZ proteins. Proteome-wide analysis of Mtb H37Rv revealed that 23.1% of expressed proteins with ≥50 amino acids contained a C-terminal PDZbm. Gene Ontology and Reactome pathway enrichment revealed their involvement in processes related to bacterial and bacterial–host interactions, including redox balance, immunomodulation, and membrane localization, at various stages of infection. Our results support the existence of a PDZbm-mediated interface between Mtb and the human host, extending the PDZbm mimicry hypothesis beyond viruses to bacterial systems as an immune evasion strategy. This work may open multiple research lines focused on experimental validation and the development of a comparative PDZbm catalogue to uncover conserved virulence mechanisms that may guide the design of host-directed therapeutics.

## 1. Introduction

Many bacteria have been found to secrete proteins, such as effectors and toxins, directly into the host cell with the potential to interfere with cell regulatory processes, either enzymatically or through protein–protein interactions (PPIs) [1].

Short linear motifs (SLiMs) are compact, modular elements typically comprising 3–10 amino acids that mediate transient yet specific interactions between signaling proteins. These motifs function by engaging conserved binding domains, such as the SH3, PDZ, or WW domains, to regulate various cellular communication processes [2].

A well-characterized class of SLiMs is the PDZ-binding motif (PDZbm), located at the C-terminus (CT) of proteins that interact with PDZ (PSD95/Discs large/Zonula occludens) domains. PDZbms are generally classified into three types on the basis of the sequences within their last four amino acids on the CT: X-S/T-X-Φ for type I, X-Φ-X-Φ for type II, and X-D/E-X-Φ for type III, where X represents any amino acid and Φ indicates a hydrophobic residue [3,4,5]. In the human proteome, it has been proposed that certain residues, mostly hydrophobic, are permissible at these positions [6].

The PDZ domain, which serves as the interaction module for PDZbm ligands, is one of the most abundant peptide-recognition domains in the human genome and is widely conserved among metazoans [2,4,6]. This globular domain is composed of five or six β-strands and two α-helices, where one strand (β2) and one helix (α2) form a cleft where the PDZbm ligand is inserted in an antiparallel β-strand conformation. PDZ domain-containing proteins (PDZ proteins) typically act as scaffolds, assembling multiprotein complexes and localizing them to specific subcellular compartments. These scaffolds are essential for the formation of protein interaction networks that regulate key cellular functions, such as signal transduction, intracellular trafficking, apoptosis, and cell polarity [5,7].

Many viruses hijack PDZ-dependent interactions by mimicking PDZbms to subvert host processes, promoting viral replication and dissemination. These mechanisms have been extensively studied in the context of viral oncogenesis [8]. We have previously proposed that viruses infecting immune cells may hijack these PDZ-mediated protein–protein interactions (PPIs) as a common strategy to evade host immune responses [9].

In contrast to viruses, bacteria, which are more complex biological entities, may use PDZ-dependent PPIs in the regulatory networks of their own metabolic and fitness functions. However, the bacterial PDZ-based interactome is poorly characterized and largely unexplored [10]. In bacterial pathogens, PDZbms proteins are enriched among effector proteins secreted via specialized systems such as the type III (T3SS) and type IV (T4SS) secretion systems [11].

*Mycobacterium tuberculosis* (Mtb), an intracellular pathogen capable of long-term persistence in macrophages, possesses a type VII secretion system (T7SS). These systems secrete a subset of effector proteins involved in virulence, affecting macrophage functions. The Mtb genome encodes five paralog T7SSs, also known as ESX systems (ESX-1 to ESX-5) [12,13].

The most extensively studied effector is the early secreted antigenic target of 6 kDa (ESAT-6, EsxA), a key virulence factor secreted into the macrophage cytoplasm during infection [14,15]. EsxA interacts with the host PDZ protein syntenin-1, presumably through its type II PDZbm (sequence GMFA) located at its C-terminus [16]. The authors discussed that this interaction may impair the scaffolding function of syntenin-1, disrupting phagosomal trafficking and contributing to Mtb immune evasion [16]. However, direct evidence that these effects are specifically mediated by the PDZbm remains limited.

EsxA and its partner CFP-10 (EsxB), both encoded by the ESX-1 locus, have paralogs in other ESX systems: EsxC and EsxD (ESX-2), EsxH and EsxG (ESX-3), EsxT and EsxU (ESX-4), and EsxN and EsxM (ESX-5), respectively [13]. These duplications and diversifications reflect the importance of ESX systems throughout the Mtb genome.

In this theoretical study, we found that six of the ten Esx effector proteins studied contained a PDZbm. Modelling of the PDZbm-dependent interaction with host syntenin-1 supported the notion that EsxA and EsxG target syntenin-1, identifying novel Mtb Esx interactors (EsxC, EsxG, and EsxN) with host PDZ proteins. Additionally, our genome-wide analysis of Mtb PDZbm-containing proteins revealed that PDZbms are present in 23.1% of the Mtb proteome, many of which are involved in diverse processes at the host–pathogen interface.

## 2. Results

### 2.1. Composition and C-Terminal Motifs of Esx Proteins

Ten secreted Esx proteins present in five ESX secretion systems (ESX-1 through ESX-5) in Mtb H37Rv were analyzed (Table 1). The protein lengths ranged from 94 to 107 amino acids. EsxM in ESX-5 lacks the PDZbm motif due to an early stop codon, whereas EsxD (Esx-2), EsxH (ESX-5), and EsxT (ESX-4) contain sequences that depart from our PDZbm definition criteria. Among the remaining six Esx proteins, four have type II PDZbms, and two have type I PDZbms (Table 1).

Using a support vector machine trained on validated human and mouse PDZ domain interactions, no sequence or structure interactors were predicted for any protein lacking a C-terminal PDZbm, consistent with the binding requirements of the motif. Among the motif-bearing proteins, 23 PDZ proteins were identified as potential interactors (Table 1), although the number of predicted interactors varied across Esx paralogs: EsxG presented the largest interaction profile (*n* = 20), suggesting potentially broad PPI engagement, followed by EsxC (*n* = 3). Coincidentally, 13 of these interactors (Table 1) were previously analyzed in macrophages and dendritic cells, nine of which were transcriptionally regulated upon stimulation with Mtb H37Ra or lipopolysaccharide in our experimental assays [18].

### 2.2. Docking Result

**Figure 1:**
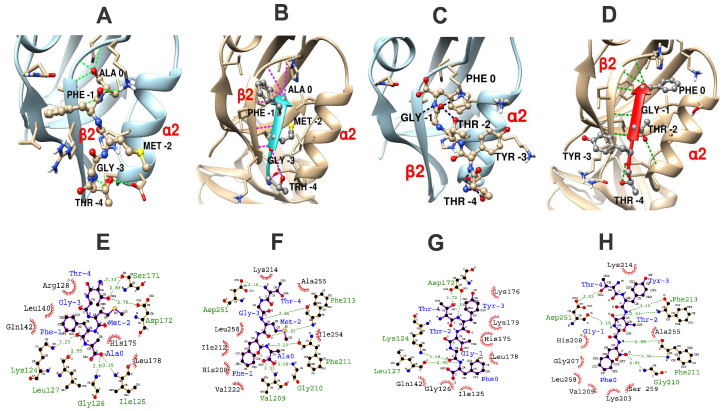
PDZbm interactions between syntenin-1 and the C-terminal pentapeptides of EsxA and EsxG. Top panel: (**A**–**D**) Molecular docking models of EsxA (**A**,**B**) and EsxG (**C**,**D**) bound to PDZ domain 1 (**A**,**C**) and PDZ domain 2 (**B**,**D**) of syntenin-1, respectively. Docking was performed using the full-length syntenin-1 structure (PDB ID: 1W9E), which has domain-specific docking boxes. The receptor and ligand chains are visualized as ribbons, and the pentapeptide residues are shown as spheres and sticks, with side chains represented as bars and labelled in black. The blue and red arrows represent the canonical PDZbm β-strand conformation. Hydrogen bonds are depicted as thin dashed lines. Only in domain 2-bound complexes (**B**,**D**) do EsxA and EsxG adopt a typical PDZbm β-strand conformation. Bottom panel: (**E**–**H**) Two-dimensional interaction diagrams generated with LigPlot, illustrating hydrogen bonds (green dashed lines) and hydrophobic contacts (red semicircles). Panels (**E**,**F**) show interactions of EsxA and EsxG with PDZ domain 1, whereas panels (**G**,**H**) show their respective interactions with PDZ domain 2. Only hydrogen bonds between the peptide ligand and syntenin-1 are displayed. Distances are in Å. Additional Esx paralog–syntenin-1 docking results are available in Supplemental Figures S1 and S2.

Syntenin-1 contains two PDZ domains and both have been reported to bind to the Mtb EsxA C-terminus with high affinity [16]. To investigate whether this interaction is dependent on the EsxA–PDZbm, we used docking analysis. To validate our docking approach, we used MDockPep to redock the known interaction [19] of the NEFYF peptide into both syntenin-1 PDZ domains (PDB: 1W9E), obtaining RMSD values of 4.590 Å for PDZ1 and 1.525 Å for PDZ2, supporting the reliability of our approach. Then, thirty models were generated per Esx interaction complex, and the best poses were selected on the basis of canonical ligand placement, where the peptide adopts an antiparallel β-strand aligned with the β2 strand of the PDZ domain [19,20]. Among the Esx proteins, the most stable complexes were EsxA–syntenin–1 PDZ2 and EsxG–syntenin–1 PDZ2 (Figure 1; Table 2). EsxT (GAWAR), despite lacking the motif, also docked to both PDZ domains with favorable scores (–93.5 and –86.2, respectively); however, the ligand was oriented perpendicularly to the binding groove, with its C-terminal tryptophan buried and the terminal arginine exposed to the solvent. Thus, EsxT failed to form the canonical PDZbm interaction with its characteristic β-strand conformation (Supplemental Figure S1), and no canonical PDZbm interaction was observed. Similarly, other Esx proteins (EsxB, C, D, H, N, and U) also docked to syntenin-1 (Table 2, Supplemental Figures S1 and S2), but none adopted an antiparallel β-strand structure. Additionally, compared with those of EsxA and EsxG, their generally lower docking scores suggest weaker or noncanonical interactions.

### 2.3. Distribution of Encoded PDZbm Proteins Across the Mtb Genome

**Figure 2:**
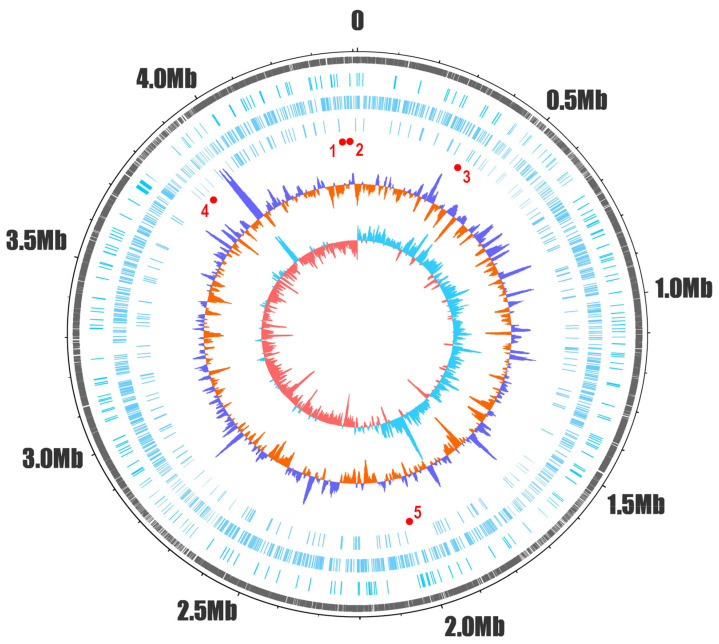
Circular representation of the Mtb genome and distribution of PDZbm-containing proteins. The outermost dashed ring displays all unique open reading frames (ORFs) in black, arranged according to their genomic positions. Concentric three rings represent the positions of genes encoding type I, type II, and type III PDZbms (blue), respectively. The following ring illustrates deviations in G + C content from the genome average, with variations above (purple) and below (orange) the genome average (65.5%). At the center, the GC skew is plotted, where positive values (light blue) indicate leading-strand bias and negative values (pink) indicate lagging-strand bias. Red dots and numbers mark approximate locations of the respective ESX (1–5) secretion system loci.

Using the Entrez API, we retrieved all 5396 proteins associated with the Mtb H37Rv reference genome, including both genome-specific (NP_, YP_) and nonredundant (WP_) accessions. After removing redundant entries, we analyzed 3896 unique protein sequences to identify C-terminal PDZbms. In total, 899 proteins (23.1%) encoded a C-terminal PDZbm. Mapping these genes onto a circular chromosome plot (Figure 2) revealed an extensive distribution around the 4.4 Mb genome. The local G + C content and GC skew appeared to be unrelated to the distribution of PDZbm-encoding genes.

In the Mtb circular genome (4.4 Mb), four of the five ESX systems are clustered within an ~1 Mb region (Figure 2). When mapped relative to the origin of replication (at zero Mb), ESX-1 and ESX-2 are located upstream at positions 4.35 Mb and 4.37 Mb, whereas ESX-3 lies just downstream at 0.35 Mb. ESX-5 is positioned at 3.86 Mb, which is still within the 1 Mb region. ESX-4, apparently the ancestral system from which the others evolved [13], is located farther away at 2.03 Mb. Whether this apparent clustering of ESX loci is related to functional or regulatory mechanisms remains to be understood. There are additional Esx-PDZbm proteins (EsxI, EsxL, EsxO, and EsxV) in the Mtb chromosome outside the T7SS loci, with EsxI and EsxV having identical protein sequences (Supplemental Data).

Notably, *ppe68*, *esxA*, *esxB*, *eccD1*, *espJ* and *espK* (Supplemental Data), which are six of the nine ORFs in the region of difference 1 (RD1), a locus in the ESX-1 system that is absent in the attenuated *M. bovis* BCG strain and known to contribute to virulence, encode PDZbms.

**Figure 3:**
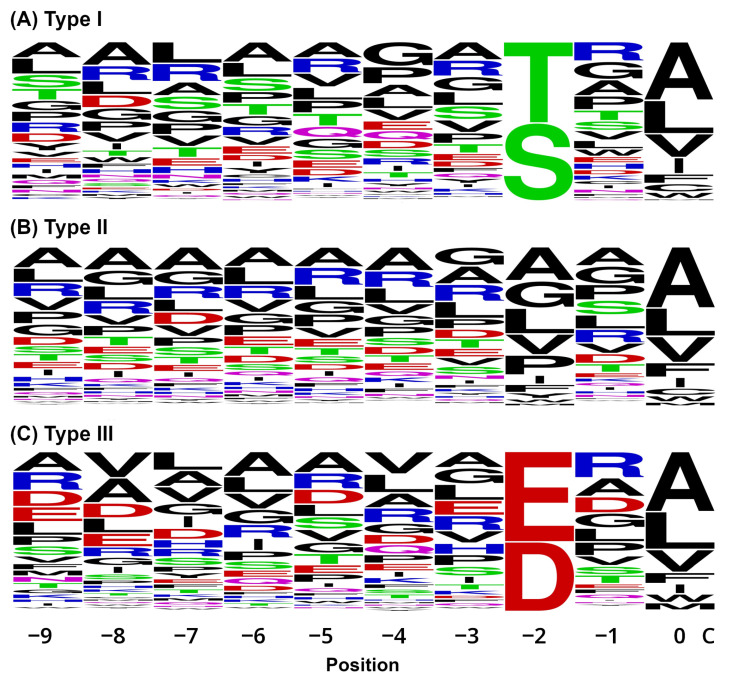
Weblogos of Mtb CT PDZbm proteins by type. Among the 3896 unique proteins, 899 (23.1%) proteins with ≥50 amino acids had a PDZbm encoded in the Mtb H37Rv genome: 147 (3.8%) were type I (**A**), 625 (16.0%) were type II (**B**), and 127 (3.3%) were type III (**C**).

To investigate whether residues upstream of the canonical PDZ-binding motif contribute to binding specificity, we analyzed sequence conservation from positions –9 to 0 relative to the C-terminus (Figure 3). Across all three types, alanine was the most dominant residue upstream (positions –3 to –9), suggesting a bias for small, helix-favoring side chains flanking the PDZbm. Arginine was the next most frequent residue, potentially playing a secondary role in modulating binding specificity or surface charge. In type I motifs, serine was enriched not only at the –2 position but also at multiple upstream sites (–3, –6, –7, and –9), which may support additional hydrogen bonding or posttranslational regulatory mechanisms. The type III motifs included aspartic and glutamic acid residues at position –2 but also at upstream positions (–3, –5, –8, and –9), suggesting the presence of similarly charged residues potentially important for electrostatic interactions with PDZ domains.

### 2.4. Gene Ontology and Pathway Enrichment Analyses

**Figure 4:**
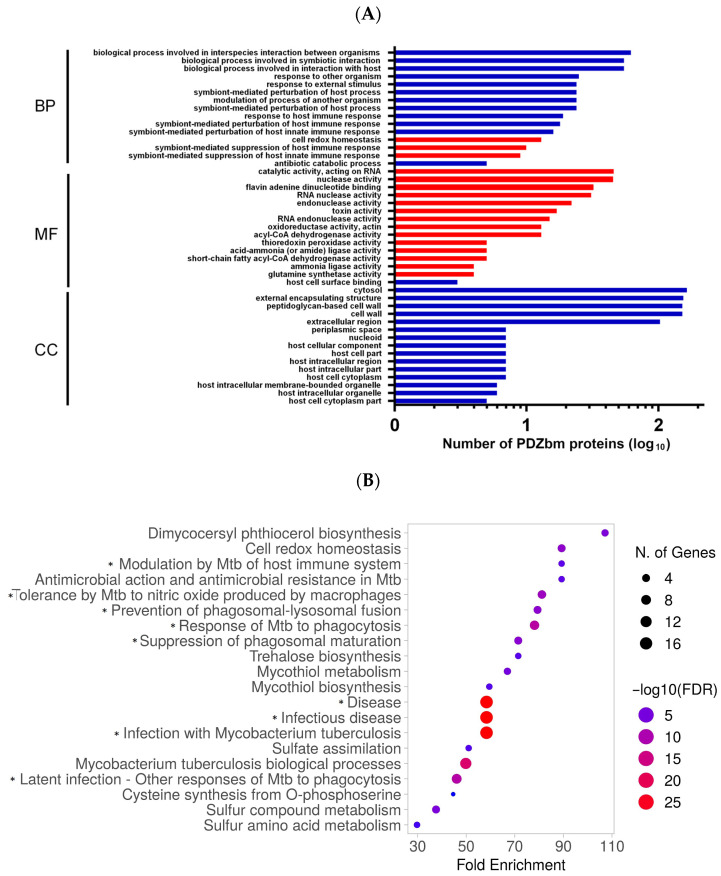
GO annotations and Reactome pathway enrichment analyses of PDZbm proteins in Mtb. (**A**) Bar plot showing the top 15 most enriched GO terms associated with PDZbm proteins via DAVID. Red bars represent statistically significant enrichment (*p* ≤ 0.05). BP: Biological Process, MF: Molecular Function, CC: Cellular Component. The *X*-axis was plotted on a logarithmic scale. (**B**) Reactome pathway enrichment analysis. The number of genes enriched in each pathway is indicated by node size; a higher –log (FDR) value means a lower false discovery rate (FDR), where red is highly significant and blue is nonsignificant. Pathways related to human–Mtb interactions are labelled with an asterisk (*).

We performed GO enrichment on the set of PDZbm-encoded proteins and selected the top 15 terms in each category for visualization (Figure 4A). In the Biological Process category, the most represented terms, although without statistical significance, were: “biological process involved in interspecies interaction between organisms,” “biological process involved in symbiotic interaction,” and “biological process involved in interaction with host.” Three biological process terms reached significance (*p* < 0.05), although with a smaller number of associated genes. These were “cell redox homeostasis” (*ahpC*, *ahpE*, *bcp*, *dlaT*, *glbN*, *lpdC*, *nrdH*, *Rv0688*, *thiX*, *tpx*, *whiB1*, *whiB2*, *whiB7*), “symbiont-mediated suppression of host immune response” (*clgR*, *eccD1*, *esxA*, *espL*, *ndkA*, *pknG*, *pstS3*, *Rv1410c*, *Rv2295*) and “symbiont-mediated suppression of host innate immune response” (*clgR*, *eccD1*, *espL*, *fadD28*, *ndkA*, *pknG*, *pstS3*, *Rv1410c*, *Rv2295*). These results point to potential roles for C-terminal PDZbm motifs in regulating oxidative stress and modulating host immune responses. In the Molecular Function category, the most represented terms were “catalytic activity acting on RNA” and “nuclease activity”, indicating that PDZbm proteins may frequently engage in nucleic acid processing. Additionally, “host cell surface binding” was also observed (involving *esxA*, *esxB*, and *Rv2004C*), although it did not reach statistical significance. For the Cellular Component category, the most frequent PDZbm localizations were “cytosol,” “external encapsulating structure,” “peptidoglycan cell wall,” “cell wall,” and “extracellular region.” The latter included several *esxA* paralogs, but again, this term failed to achieve statistical significance.

Reactome pathway analysis (Figure 4B) was ranked by fold enrichment, PDZbm count and false discovery rate (FDR). This analysis provided insights into the Mtb pathways; among the most represented pathways were those associated with Mtb biological processes, such as trehalose, mycothiol, and dichycocersyl phthiocerol biosynthesis and mycothiol, sulfur compound and sulfur amino acid metabolism. Additionally, we focused on pathways directly related to human–Mtb interactions. Among the most enriched genes were “Disease,” “Infectious disease,” and “Infection with *Mycobacterium tuberculosis*,” all of which shared a common subset of PDZbm-encoding genes (*ahpC*, *ahpE*, *bfrB*, *dlaT*, *eis*, *esxA*, *esxG*, *glbN*, *lpdC*, *mscR*, *ndkA*, *rpoA*, *rpoB*, *Rv1410c*, *Rv2779c*, *Rv3655c*, *tpx*).

In addition to those broad categories, multiple Reactome pathways specifically related to host–pathogen interactions, immune regulation, redox balance, and phagosome maturation arrest were enriched, such as “Modulation by Mtb of host immune system” (*esxA*, *Rv2779c*), “Tolerance by Mtb to nitric oxide produced by macrophages” (*ahpC*, *ahpE*, *glbN*, *mscR*, *tpx*), “Prevention of phagosomal–lysosomal fusion” (*esxG*, *lpdC*, *ndkA*, *Rv1410c*), “Response to Mtb phagocytosis,” (*eis*, *esxA*, *esxG*, *lpdC*, *ndkA*, *Rv1410c*, *Rv3655c*), “Suppression of phagosomal maturation” (*esxG*, *lpdC*, *ndkA*, *Rv1410c*), and “Latent infection–Other responses of Mtb to phagocytosis,” (*ahpC*, *ahpE*, *bfrB*, *dlaT*, *glbN*, *lpdC*, *mscR*, *tpx*). Complete gene lists are available in the Supplemental Data.

**Figure 5:**
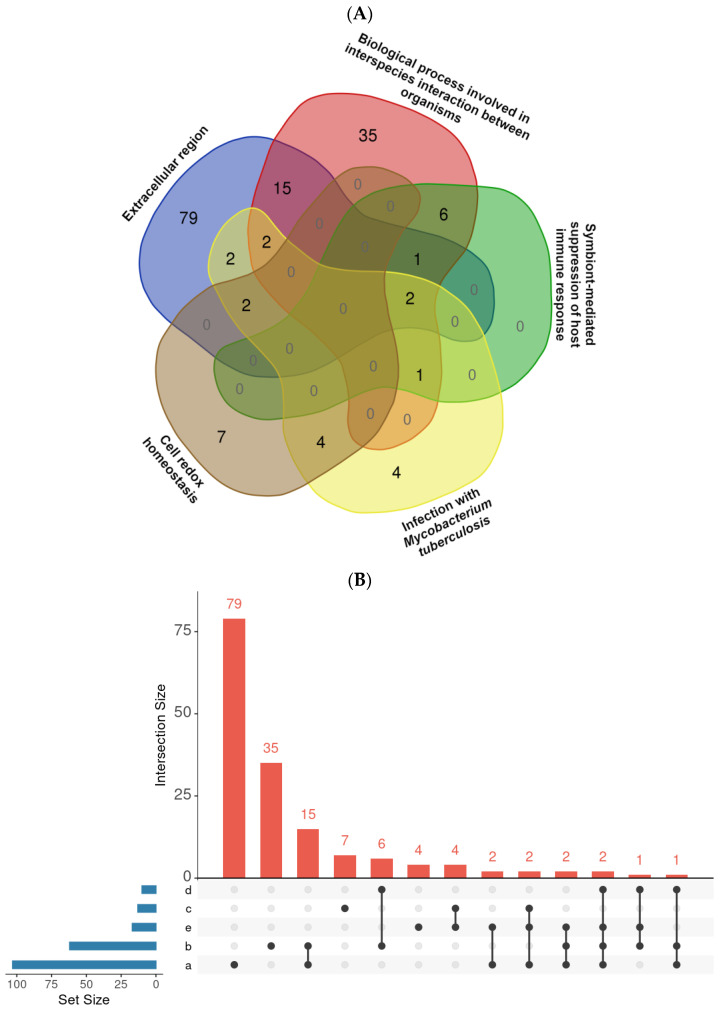
Overlap of GO and Reactome annotation categories among PDZbm-bearing proteins. (**A**) Venn diagram representation of five interacting categories; numbers represent proteins in one or more categories. (**B**) Upset plot showing the total number of proteins in each category (horizontal bars, which correspond to a: extracellular region, b: biological process involved in interspecies interaction between organisms, c: cell redox homeostasis, d: symbiont-mediated suppression of host immune response, and e: infection with *Mycobacterium tuberculosis*) and in shared categories (vertical bars with the respective number of proteins).

To assess the relationships between the most relevant GO annotations and Reactome pathways, the overlapping PDZbm proteins were identified (Figure 5). Among the interactions, those in which we found Esx effectors are highlighted. A total of two PDZbm coding genes (*esxA* and *ndkA*) overlapped between “Extracellular region,” “Infection with *Mycobacterium tuberculosis*,” “biological process involved in interspecies interaction between organisms,” and “symbiont-mediated suppression of host immune response.” *esxG* and *BfrB* overlapped between the “Extracellular region” and “Infection with *Mycobacterium tuberculosis*.” Complete lists of PDZbm genes can be accessed in the Supplemental Data.

## 3. Discussion

Pathogenic bacteria interact with host cells through effector proteins delivered via specialized secretion systems. These effectors often contain structural motifs that mimic host protein components (molecular mimicry), allowing pathogens to hijack cellular processes by selectively tuning specific host functions. Two major types of hijacking via protein mimicry can be described: globular domains and SLiMs [1,21]. The PDZbm SLiMs are recognized as virulence determinants of many viral proteins. However, the role of bacterial PDZbms has been comparatively less explored. A decade ago, Yi et al. [11] demonstrated that bacterial effectors secreted via the T3SS and T4SS secretory systems are enriched in PDZbm proteins and discussed how these proteins are believed to have evolved to exploit the host PDZ interactome, thereby increasing bacterial pathogenicity.

Mtb employs a specialized T7SS, which is essential for pathogenesis and immune evasion [22,23,24]. In this work, we identified at least six T7SS Esx effectors with PDZbms among the ten studied within the T7SS loci. Our docking analyses support previous findings [16], suggesting that syntenin-1 is a likely target of EsxA; similarly, we found that EsxG may be another interactor. EsxA and EsxG have been implicated in the arrest of the immune response [14,25,26,27]. Syntenin-1 is a key regulator of membrane dynamics and participates in regulating immune signaling through various PDZ-dependent PPIs, contributing to vesicle trafficking, exosome biogenesis, and cell migration [28,29,30]. Schumann et al. [16] demonstrated an interaction between EsxA and syntenin-1 via yeast two-hybrid and immunoprecipitation assays, where only EsxA, not the PDZbm-deficient paralog EsxT, interacted with syntenin-1. Our docking results corroborate that EsxA shows canonical PDZbm binding to syntenin-1, whereas EsxT fails to adopt a β-strand conformation compatible with PDZ engagement, reinforcing the validity of our model.

Syntenin-1 is exploited by various viruses that rely on endosomal entry, including SARS-CoV-2 [31], as shown by reduced infection when an inhibitory peptide that binds to the first PDZ domain of syntenin-1 is present. A pathogenic mechanism in Mtb is dysregulation of the endocytic pathway via disruption of phagosome–lysosome fusion in macrophages, which circumvents lysosomal degradation and facilitates intracellular survival. EsxA plays a key role in this disruption by inhibiting phagolysosomal maturation, possibly by interacting with syntenin-1 [14,15]. In syntenin-1 knockout mice, Firmicutes and immunoglobulin levels were increased in feces [32], suggesting that syntenin-1 may play a role in bacterial control. Further studies should experimentally investigate the canonical PDZ-dependent interactions of EsxA and EsxG with syntenin-1 and possibly other PDZ proteins.

Mtb translocation from phagosomes to the cytosol is a critical virulence mechanism and is known to be dependent on EsxA [33,34]. A single-point mutation, M93T, corresponding to the PDZbm position −2 (M93T; EsxAmut) abolishes phagosomal escape and reduces virulence [35]; thus, EsxA-dependent phagosomal escape requires its type II PDZbm. Our docking results indicate that EsxAmut fails to form an antiparallel β-strand structure and has lower docking scores than EsxA and EsxG (Table 2), suggesting weaker interactions. The PDZ-dependent interaction of EsxA–syntenin-1 might represent a mechanism for virulence and immune evasion that requires experimental confirmation.

Our modelling also revealed that EsxG interacts with an extended set of host PDZ proteins. Notably, nine of the PDZ proteins identified as potential Esx-PDZbm targets in our predictive screen (Table 1) overlapped with those previously reported to be transcriptionally regulated in macrophages and dendritic cells upon stimulation with Mtb H37Ra or lipopolysaccharide [18]. This observation suggests a relevant involvement of such PDZ proteins in the immune response and experimental demonstration of Esx protein interactions would be important to understand their role for Mtb virulence.

We found that 23% of the Mtb proteome contains PDZbms that are distributed across the genome and are not restricted to operons or pathogenicity islands (Figure 2). This significant percentage of proteins suggests that PDZbms contribute to multiple bacterial metabolic and functional processes throughout the bacterial cell such as “RNA endonuclease activity” or “mycothiol biosynthesis” (Figure 4A and Supplemental Data), and also to bacterial–host interaction (Figure 4A). For example, the Esx pairs EsxA/EsxB and EsxG/EsxH display distinct surface features and expression profiles, indicating distinct functional roles, with EsxA/EsxB being implicated in binding to cell surface receptors and pathogen–host cell signaling [36,37], and EsxG/EsxH in iron and zinc acquisition and the impairment of antigen presentation [25,26], the latter being a clear example of PDZbm-bearing proteins involved in both bacterial and bacterial–host processes.

In general, Mtb PDZbm proteins appeared to be involved in various stages of infection, such as the response to Mtb phagocytosis and latent infection (Figure 4B). In addition to Esx effectors, Mtb PDZbm proteins are involved in immune evasion by modulating macrophage autophagy (Eis), inflammatory responses (Eis), and cell death via a reactive oxygen species (ROS)-dependent pathway (bfr, Eis, Ndk) (Figure 5, Supplemental Data) [38,39].

Interestingly, several PE and PPE proteins, comprising approximately 7–10% of the Mtb proteome, exhibit structural and secretion mechanisms with the Esx family [40]. These proteins are known to participate in host–pathogen interactions and facilitate nutrient transport across the mycobacterial outer membrane, often being secreted as heterodimers via the T7SS [40]. We identified 24 PE and 6 PPE proteins containing PDZbms, including PPE68 (secreted by ESX-1), PE15 (an ESX-3 substrate), and PPE36, which are implicated in iron homeostasis (Supplemental Data).

Pathogen mimicry is often “imperfect,” allowing enough structural similarity for binding while avoiding definitive interactions. This enables subtle manipulation of host pathways, such as vesicular trafficking, apoptosis, or immune signaling [21]. However, PDZbm–PDZ interactions involve a complex interplay of factors. While a single PDZbm can potentially engage multiple PDZ domains, the affinity and specificity of these interactions are highly context dependent and shaped by the core motif sequence, upstream residues, local conformation, and even posttranslational modifications such as phosphorylation [4,41,42,43], where even point mutations can significantly shift motif interactions [42,43]. Notably, our sequence logo analysis (Figure 3) revealed conservation at upstream positions (−3, −5, and −7), in line with previous studies showing that these residues contribute to binding specificity and affinity [41,43].

The sequences of syntenin-1 have been highly conserved for millions of years. Conversely, the C-terminal regions of Mtb Esx paralogs display mutations across nearly all positions (Table 1). The evidence suggests that viral PDZbms have evolved to target several PDZ proteins simultaneously, enabling them to manipulate multiple cellular pathways and conferring an evolutionary advantage, in contrast with endogenous sequences, which tend to be more specific [9,43,44]. The promiscuity of PDZbms, particularly in viruses such as HPV16 and SARS-CoV-2, maintained similar binding affinities with smaller changes in ΔG after mutations were introduced [43], which may facilitate immune evasion and adaptation to diverse hosts [43,45,46]. This broad targeting could facilitate rapid integration into the host cellular machinery, supporting processes such as proliferation and immune evasion, thereby favoring pathogen survival. Whether similar mechanisms occur in bacterial systems still needs to be characterized.

For our modelling and docking analyses, we focused on short CT peptides containing the canonical PDZbm and used human-defined PDZbm criteria to select bacterial proteins for further study. This approach may overlook additional determinants present elsewhere in the ligand proteins or in the full-length proteins, and may miss bacterial proteins capable of adopting a canonical PDZ-PDZbm conformation. The docking calculations were conducted specifically to determine which ligands adopted the antiparallel β-strand canonical conformation, which serves as the primary criterion for a canonical PDZbm interaction [20]. Additionally, the ITScores provided a useful assessment for the differential interaction energy between syntenin-1 and the ligands. However, these docking results should be interpreted with caution and regarded as a hypotheses-generating approach that requires validation through molecular dynamics studies and experimental confirmation. Further, the GO terms and Reactome pathways shown (Figure 4 and Figure 5) are unable to distinguish the type of molecular interaction involving the bacterial protein or clarify whether the PDZbm is involved. Currently, there is limited experimental data about this.

The accumulation of evidence suggests that Mtb manipulates the host PPI landscape through secreted effectors, with functional consequences that enhance infection. Our findings align with recent systems biology efforts aiming to map the Mtb–host interactome [47].

Some reports have used CT tags blocking PDZbm access, which limits the detection of native interactions [16,47]. In view of our findings, avoiding CT modifications and prioritizing assays that preserve native motif accessibility would enable quantitative and high-throughput analysis of PDZ domain interactions [48].

Experimental validation of PDZ-PDZbm interactions in pathogens is still limited [6,48]. Our findings highlight the diverse roles of PDZbm-containing proteins in Mtb virulence and emphasize the importance of characterizing the host PDZ interactome during infection. These results expand our hypothesis [9] that PDZ-dependent viral hijacking of the immune response as a mechanism of immune evasion may also be a strategy in bacterial pathogens.

A comprehensive mapping of the Mtb PDZ interactome, focused on C-terminal interactions, could elucidate how secreted effectors manipulate host scaffolding networks. The development of a comparative catalogue of human PDZbms, including their upstream sequences, alongside those in Mtb and other pathogens, may reveal patterns of molecular mimicry and convergent evolution. Such a database would facilitate the identification of conserved PDZbm architectures shared across unrelated pathogens, revealing shared host vulnerabilities. These insights could guide the development of broad-spectrum inhibitors targeting PDZ-mediated interactions essential for pathogenicity, as shown previously [31]. With the rise of antibiotic-resistant strains, understanding and targeting the host–pathogen interface offers a promising path for next-generation therapies.

## 4. Materials and Methods

### 4.1. Protein Databases

CT sequences of Esx proteins encoded within the five recognized T7SS loci (ESX-1–ESX-5) were retrieved from UniProt. The human syntenin-1 protein, also known as syndecan-binding protein (SDCBP) or melanoma differentiation-associated gene 9 (MDA-9), contains two PDZ domains bound to NEFYF ligands in the available protein crystallographic structure (PDB ID: 1W9E) used as a receptor for docking analyses.

Protein sequences associated with *Mycobacterium tuberculosis* H37Rv (genome accession NC_000962.3) were retrieved via the following NCBI Entrez Programming Utilities [49]: the genome was queried via ESearch, linked protein records were identified via elink, and sequences were downloaded with efetch. This approach retrieves curated genome-specific, predicted genome-specific, and nonredundant protein sequences shared among multiple taxa (accessions prefixed as NP_, YP_, and WP_, respectively), resulting in a comprehensive protein set. Redundant sequences were collapsed, retaining a single representative (preferentially an NP_ or YP_ record) for each amino acid sequence.

For all three PDZbm types, position 0 (C-terminus) was limited to hydrophobic amino acids (alanine, cysteine, isoleucine, leucine, methionine, phenylalanine, tryptophan, and valine), whereas positions −1 and −3 could accommodate any amino acid. Notably, position −2 was restricted for type I PDZbms to serine or threonine, for type II PDZbms to hydrophobic residues (alanine, glycine, isoleucine, leucine, methionine, phenylalanine, proline, tryptophan, tyrosine, and valine), and for type III PDZbms to aspartic or glutamic acid. We defined these residues on the basis of a consensus from published literature [3,4,6]. A custom script, using Biopython v1.84 [50], available from Zenodo [51], was developed to filter FASTA protein sequences on the basis of the last four C-terminal amino acids for PDZbm types I–III, with an output of the last ten amino acids of each protein.

### 4.2. Docking Analyses

Previous studies using protein overlay, surface plasmon resonance, yeast two-hybrid and copurification techniques have demonstrated an interaction between EsxA and syntenin-1 [16], presumably through a PDZbm interaction. Here, we analysed the binding of nine Esx paralog proteins and an EsxA mutant (TGTFA; referred to as EsxAmut) to syntenin-1 via molecular docking. The simulations targeted each of the two PDZ-binding cavities formed by the conserved GLGF loop, the β2 strand, and the α2 helix. Peptides representing the C-terminal pentapeptides of each Esx paralog and EsxAmut were modelled in a linear, extended β-antiparallel conformation via the UCSF ChimeraX website [52,53]. Docking was performed in triplicate via the MDockPep web application [54,55]. The best-fitting poses were selected on the basis of their alignment with the binding cavity formed by the conserved GLGF loop, the β2 strand, and the α2 helix of human syntenin-1, as observed in various type II PDZbms [19,20]. Protein–ligand interaction diagrams were generated with ChimeraX and LigPlot [56].

### 4.3. Human Protein Binding Prediction

A sequence- and structure-based interaction algorithm was used to predict human PDZ-Mtb protein-dependent interactions [17] with Esx-secreted proteins. The model is based on a support vector machine trained with experimentally validated interactions from human and mouse proteomes.

### 4.4. Sequence Representations

PDZbm sequence patterns were visualized via the WebLogo 2.8.2 application [57], with the relative frequency of each amino acid indicated by the height of each amino acid symbol. DNAPlotter [58] was used to represent the distribution of PDZbm-encoding genes in the circular Mtb genome.

### 4.5. Gene Ontology and Pathway Enrichment Analyses

Gene Ontology (GO) and pathway enrichment analyses of the PDZbm gene sets were performed via the Database for Annotation, Visualization and Integrated Discovery (DAVID) v2023q4 [59,60]; subsequently, a bar graph was generated in GraphPad Prism v.10.4.2 for Windows for enrichment analysis, including biological process (BP), molecular function (MF) and cellular component (CC) analyses. REACTOME pathway analysis was performed on the ShinyGO 0.82 enrichment analysis server [61]. A resulting Venn diagram was created [62] with relevant GO terms and pathways.

## Figures and Tables

**Table 1:** Mtb Esx paralogs and their putative human ligands. The Esx proteins present in the Mtb ESX1-5 systems are described. a: Interactions were predicted in POW [17]. PDZ gene mRNA expression was modified with H37Ra or lipopolysaccharide stimulation in macrophages (b), dendritic cells (c) or both (d), as shown previously [18].

Esx Protein	UniProt Acc. Number	Length (aa)	C-Terminus Sequence (PDZbm Type)	Human PDZ-Protein Putative Interactor		
				Experimental	Modelled by Sequence ^a^	Modelled by Structure ^a^
EsxA (ESAT6)	P9WNK7	95	VTGMFA (2)	Syntenin-1 ^d^		
EsxB (CFP10)	P9WNK5	100	LSQMGF (2)	Syntenin-1 ^d^		
EsxC	P9WNI1	95	AIAGLF (2)		MPDZ SHANK2	DVL2 ^d^
EsxD	O05453	107	GASHGS (none)			
EsxG	O53692	97	STYTGF (1)		GRD2I MAGI3 ^c^ MAGIX MAST1 MAST2 ^d^ PDZD3 PDZK1 RGS12 RHPN1 SHANK1 SHANK2 SHANK3 SLC9A3R1 ^d^ SLC9A3R2 ^b^ SNX27 ^c^	DVL1 DVL2 ^d^ GRIP1 GRIP2 RAPGEF6 ^b^ SHANK3 SLC9A3R2 ^b^
EsxH	P9WNK3	96	AAKWGG (none)			
EsxM	--	-	Early stop codon			
EsxN	P9WNJ3	94	VGSSWA (1)		INADL ^c^ MPDZ	
EsxT	I6YC53	100	AGAWAR (none)			
EsxU	I6Y3I6	105	AAGGDL (2)		RHPN1	MAGI3 ^c^

**Table 2:** Binding analysis between each syntenin-1 PDZ domain and the C-terminal Esx paralogs. The ITScore, a statistical mechanics-based energy scoring function, was used to assess each complex on the basis of structural patterns observed in experimentally determined protein structures. The number of hydrogen bonds and hydrophobic interactions was the same as that observed in the selected pose. Lower scores indicate more stable and plausible binding conformations. Proteins are listed based on the best ITScore for Domain 1 binding.

Paralog Protein	C-Terminus Sequence	ITScore		Hydrogen Bonds		Hydrophobic Interactions	
		Domain 1	Domain 2	Domain 1	Domain 2	Domain 1	Domain 2
EsxA	TGMFA	−86.2	−84.8	7	6	5	7
EsxG	TYTGF	−85.0	−96.7	4	7	7	8
EsxC	IAGLF	−84.6	−88.6	4	5	6	7
EsxB	SQMGF	−81.0	−87.0	3	7	7	6
EsxAmut	TGTFA	−78.9	−70.2	6	4	4	7
EsxN	GSSWA	−74.3	−73.6	7	7	5	7
EsxU	AGGDL	−53.1	−55.5	8	6	5	8
Non-PDZbm							
EsxT	GAWAR	−93.5	−86.2	2	1	11	10
EsxH	AKWGG	−80.9	−75.1	5	4	6	6
EsxD	ASHGS	−57.4	−62.4	6	8	4	3

## Data Availability

The original data presented in the study are openly available in Zenodo at https://doi.org/10.5281/zenodo.17517600 and in Figshare at https://doi.org/10.6084/m9.figshare.30524351. The Python script (version v20200505) to process the proteome data can be downloaded from Zenodo at https://doi.org/10.5281/zenodo.15757961.

## Supplementary Materials

The supporting information can be downloaded at: https://doi.org/10.6084/m9.figshare.30442814.

## Abbreviations

The following abbreviations are used in this manuscript:

SLiMs	Short linear motifs
PDZ	PSD95/Discs large/Zonula occludens domain
PDZbm	PDZ-binding motif
Mtb	*Mycobacterium tuberculosis*
T7SS	Type VII secretion system
